# The Vienna Prediction Model for identifying patients at low risk of recurrent venous thromboembolism: a prospective cohort study

**DOI:** 10.1093/eurheartj/ehad618

**Published:** 2023-09-28

**Authors:** Paul A Kyrle, Lisbeth Eischer, Hana Šinkovec, Paul Gressenberger, Thomas Gary, Marianne Brodmann, Georg Heinze, Sabine Eichinger

**Affiliations:** Division of Hematology and Hemostasis, Department of Medicine I, Medical University of Vienna, Vienna A-1090, Austria; Karl Landsteiner Institute of Thrombosis Research, Vienna A-1020, Austria; Division of Hematology and Hemostasis, Department of Medicine I, Medical University of Vienna, Vienna A-1090, Austria; Center for Medical Statistics, Informatics and Intelligent Systems, Institute of Clinical Biometrics, Medical University of Vienna, Vienna A-1090, Austria; Division of Angiology, Department of Medicine, Medical University of Graz, Graz A-8010, Austria; Division of Angiology, Department of Medicine, Medical University of Graz, Graz A-8010, Austria; Division of Angiology, Department of Medicine, Medical University of Graz, Graz A-8010, Austria; Center for Medical Statistics, Informatics and Intelligent Systems, Institute of Clinical Biometrics, Medical University of Vienna, Vienna A-1090, Austria; Division of Hematology and Hemostasis, Department of Medicine I, Medical University of Vienna, Vienna A-1090, Austria; Karl Landsteiner Institute of Thrombosis Research, Vienna A-1020, Austria

**Keywords:** Venous thromboembolism, Recurrence, Vienna Prediction Model

## Abstract

**Background and Aims:**

Patients with unprovoked venous thromboembolism (VTE) have a high recurrence risk, and guidelines suggest extended-phase anticoagulation. Many patients never experience recurrence but are exposed to bleeding. The aim of this study was to assess the performance of the Vienna Prediction Model (VPM) and to evaluate if the VPM accurately identifies these patients.

**Methods:**

In patients with unprovoked VTE, the VPM was performed 3 weeks after anticoagulation withdrawal. Those with a predicted 1-year recurrence risk of ≤5.5% were prospectively followed. Study endpoint was recurrent VTE over 2 years.

**Results:**

A total of 818 patients received anticoagulation for a median of 3.9 months. 520 patients (65%) had a predicted annual recurrence risk of ≤5.5%. During a median time of 23.9 months, 52 patients had non-fatal recurrence. The recurrence risk was 5.2% [95% confidence interval (CI) 3.2–7.2] at 1 year and 11.2% (95% CI 8.3–14) at 2 years. Model calibration was adequate after 1 year. The VPM underestimated the recurrence risk of patients with a 2-year recurrence rate of >5%. In a post-hoc analysis, the VPM’s baseline hazard was recalibrated. Bootstrap validation confirmed an ideal ratio of observed and expected recurrence events. The recurrence risk was highest in men with proximal deep-vein thrombosis or pulmonary embolism and lower in women regardless of the site of incident VTE.

**Conclusions:**

In this prospective evaluation of the performance of the VPM, the 1-year rate of recurrence in patients with unprovoked VTE was 5.2%. Recalibration improved identification of patients at low recurrence risk and stratification into distinct low-risk categories.

## Structured Graphical Abstract

**Structured Graphical Abstract:**
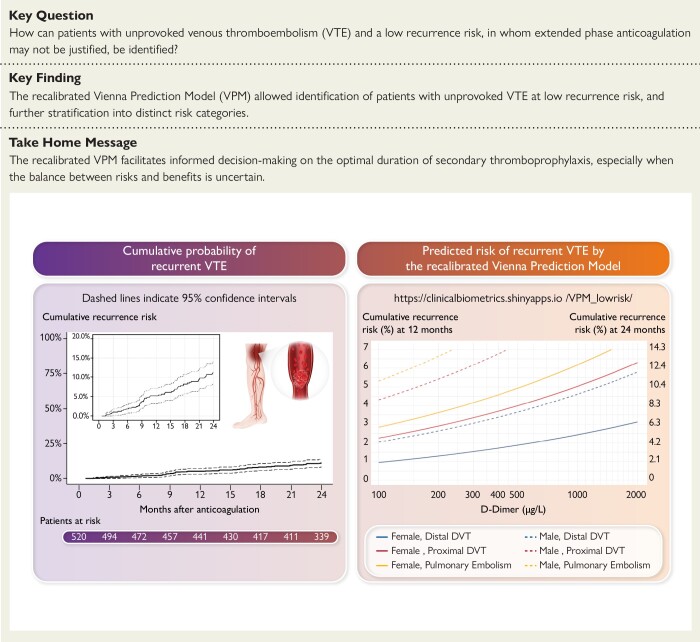
The left part of the figure shows the cumulative probability of recurrence in patients with unprovoked VTE and a low risk identified by the VPM. The right part of the figure shows the predicted risk of recurrent VTE by the recalibrated VPM, which can be assessed by a web-based calculator (https://clinicalbiometrics.shinyapps.io/VPM_lowrisk/). DVT, deep-vein thrombosis; VTE, venous thromboembolism; VPM, Vienna Prediction Model.

**See the editorial comment for this article ‘The science and art of predicting recurrentVTE’, by M. Carrier and P. Verhamme, https://doi.org/10.1093/eurheartj/ehad712.**

## Introduction

Venous thromboembolism (VTE) is a potentially fatal disease with an annual incidence of 1–2 per 1000 persons.^1^ The most common site of VTE is deep-vein thrombosis (DVT) of the leg, which causes pulmonary embolism when the thrombus dislocates. Venous thromboembolism tends to recur. Patients with VTE provoked by a transient risk condition have a lower recurrence risk^2–8^ than patients with a persisting risk provoked by cancer, inflammatory bowel disease, or antiphospholipid antibodies.^9–11^ Patients with VTE in the absence of a risk condition, so-called unprovoked VTE, are within the highest recurrence risk category.^12–15^ In a systematic review, these patients had a 5-year recurrence risk of 25% with a case fatality rate of 4%.^16^ Consequently, expert panels suggest offering extended-phase anticoagulation to patients with unprovoked VTE.^17–19^ However, many patients will never experience recurrence while being exposed to a bleeding risk. According to a meta-analysis, the incidence of major bleeding during extended-phase anticoagulation is considerable with 1.74 events per 100 person-years for vitamin K antagonists and 1.12 events per 100 person-years for the direct oral anticoagulants.^20^ There are some arguments that question the concept of extended-phase anticoagulation for all patients with unprovoked VTE: (i) the follow-up in trials may not have been long enough to indisputably determine that the benefits of extended-phase anticoagulation outweigh the bleeding risk, (ii) the findings of trials may not reflect the risks and benefits of extended-phase anticoagulation in routine care, and (iii) the benefit from extended-phase anticoagulation may not be large enough to justify the burden and costs of long-term therapy. Therefore, identifying patients at low recurrence risk who may be candidates for limited duration of anticoagulation is of utmost clinical importance. Prediction models based on clinical and laboratory variables that had been evaluated in large-scale clinical studies look promising in achieving this goal.^21–29^ One of them, the Vienna Prediction Model (VPM), estimates the probability of recurrence in patients with an unprovoked VTE by integrating sex, thrombosis site, and D-dimer.^29^ In the original VPM, two-thirds of the patients with unprovoked VTE had a recurrence risk of <5% within 1 year after anticoagulation withdrawal. We externally validated the model by using a pooled individual patient database and confirmed the ability of the VPM to stratify patients according to their recurrence risk.^30^ The analysis also revealed that after 1 year the predicted cumulative recurrence rates tended to underestimate the observed cumulative rates. In a study from the Netherlands, the clinical impact of the VPM on reducing the recurrence risk in patients with unprovoked VTE was compared with usual care.^31^ The VPM showed good discriminative performance with a *c*-statistic of 0.76. Again, the model underestimated the recurrence risk, particularly above a threshold of 5%.

In 2013, we initiated a prospective cohort study in patients with unprovoked VTE and a low recurrence risk estimated by the VPM. The first objective was to assess the performance of the VPM in identifying patients with a low recurrence risk. The second objective was to evaluate if updating the VPM improved its performance.

## Methods

### Patients and study design

We performed a prospective cohort study at the Division of Hematology and Hemostasis, Department of Medicine I, Medical University of Vienna, and at the Division of Angiology, Department of Medicine, Medical University of Graz, both Austria (clinical trial registration NCT01972243). The study was approved by the ethics committees of both institutions and was conducted according to the Declaration of Helsinki. All patients gave written informed consent.

Patients with an objectively diagnosed symptomatic DVT of the leg and/or symptomatic pulmonary embolism were eligible. Patients were included if they were 18 years of age or older, were treated with an oral anticoagulant for at least 3 but not longer than 7 months, and had no reason for long-term anticoagulation other than VTE. We excluded patients with a history of VTE; muscle vein thrombosis; VTE associated with surgery, trauma, pregnancy, active cancer, and immobilization (defined as 75% of daytime bedridden for more than 3 days); oestrogen use within 3 months prior to VTE; and known laboratory thrombophilia (deficiency of antithrombin, protein C, or protein S, homozygosity or double heterozygosity of factor V Leiden or the prothrombin mutation, and presence of antiphospholipid antibodies).

D-dimer was measured by a quantitative immunoassay 3 weeks after anticoagulation had been discontinued. At that time, the VPM risk assessment was performed using a web-based calculator. Patients with a VPM risk score of more than 180 points which corresponds to a predicted 1-year recurrence risk of more than 5.5% were informed about their high recurrence risk. Their anticoagulant management and follow-up were left at the discretion of their physician.

Patients with a VPM risk score of 180 points or less did not resume anticoagulation and were followed prospectively. They were informed on signs and symptoms of VTE and were instructed to contact their physician or the study centre if such signs or symptoms occurred. Women were instructed to refrain from using female hormones. All patients were seen in person after 3, 12, and 24 months. At baseline, a compression ultrasound of both legs was performed to obtain reference imaging. In risk situations, thromboprophylaxis was performed according to guidelines. All other treatments that might have influenced the recurrence risk including compression stockings, antiplatelet therapy, or statins were left at the discretion of the physician.

### Diagnosis of incident venous thromboembolism

The diagnosis was established by compression ultrasound, venography, spiral computed tomography, or lung scan according to published criteria.^29^ Of note, the diagnosis of isolated distal DVT was only made when the thrombus was in the tibial anterior, peroneal, or tibial posterior veins. Patients with both pulmonary embolism and DVT were classified as pulmonary embolism.

### Outcome measures

The study endpoint was objectively confirmed symptomatic DVT of the leg or fatal or symptomatic non-fatal pulmonary embolism. Recurrent DVT was diagnosed by compression ultrasound.^32^ There had to be a new non-compressible venous segment or an increase of 4 mm or more in thrombus diameter with compression or an extension in length. If the compression ultrasound was negative or non-diagnostic and there was clinical suspicion of DVT, a compression ultrasound was repeated within 1 week.

The diagnosis of recurrent pulmonary embolism was established by spiral computed tomography or lung scan. There had to be an intraluminal filling defect in at least one segmental or larger artery or a segmental perfusion defect with normal ventilation, i.e. a ventilation perfusion mismatch. Fatal pulmonary embolism had to be diagnosed by autopsy or classified by death that could not be attributed to a documented cause and for which pulmonary embolism could not be ruled out. The diagnosis of recurrent VTE was adjudicated by an independent clinician and by a radiologist.

### Statistical analysis

In our previous study,^29^ the observed cumulative recurrence risk at 1 and 2 years among patients with a VPM risk score of 180 points or less was 4.4% (95% CI 2.7–6.2) and 8.3% (95% CI 5.7–10.7). Sample size calculation was performed based on an official communication of the Scientific and Standardization Committee of the International Society on Thrombosis and Haemostasis that cohort studies should be powered to exclude a 1-year recurrence risk of 8%.^33^ By simulating 1000 trials consisting of 500 patients with a VPM risk score of 180 points or less, who were randomly selected with replacement, we calculated that a sample size of 500 patients was needed to provide a 92.2% (simulation standard error 0.8%) power at a one-sided significance level of 2.5% to reveal that the cumulative recurrence risk at 1 year was <8%. The study had 90.5% (standard error 0.9%) power at the same significance level to detect a cumulative recurrence risk at 2 years <13%.

Baseline characteristics were described by medians and interquartile ranges for continuous variables and by absolute frequencies and percentages for categorical variables. Patients were followed from the day of discontinuation of anticoagulation until recurrent VTE, reinitiation of anticoagulation, completed follow-up of 24 months, or until they were lost to follow-up, whichever came first. We treated observations as censored if no recurrence occurred and estimated the cumulative incidence of recurrence by the Kaplan–Meier method, along with 95% confidence intervals. The recurrence rate in the second year of follow-up was estimated with the actuarial method.^34^

We tested the null hypothesis that the 1-year cumulative recurrence risk is ≥8% against the alternative hypothesis that it is <8%. We performed a single-sample *z* test based on a normal approximation at a one-sided significance level of 0.025. We tested the null hypothesis that the 2-year cumulative recurrence risk is ≥13%. A data safety monitoring board reviewed all incidences at pre-defined intervals and evaluated the study based on a priori agreed stopping rules.

Calibration was assessed by comparing observed and mean predicted cumulative incidence of recurrence at 2 years. We estimated the calibration slope by fitting a Cox regression model to the study cohort in which the linear predictors from the VPM (the sum of predictors multiplied by their predictor weights as defined by the VPM) were included as a single covariate. We computed the ratio of observed events and the number of events that were predicted by the VPM (O/E ratio) to assess general under- or overestimation. We also evaluated discrimination of the VPM in the study cohort by computing Uno’s concordance index (*c*-statistic).^35^

As post-hoc analysis, we evaluated whether analysis of the study cohort’s recurrence profile suggested modifying the weights assigned to each predictor by the VPM. We first estimated a Cox regression model including the covariates of the VPM, i.e. sex, location of index VTE, and log-base-2 transformed D-dimer, while considering the linear predictors of the VPM as an offset, i.e. as a covariate with a fixed predictor weight of 1. Rather than re-estimating new predictor weights, this model suggests how the predictor weights of the VPM should be modified to provide an ideal fit in the study cohort. We used a likelihood ratio test to test whether any of these suggested predictor weight modifications was different from 0 and should be updated. To recalibrate the model, we left the predictor weights of the VPM unchanged but re-estimated the baseline hazard function. Ninety-five percent confidence intervals were obtained by the bootstrap percentile method, re-estimating the baseline hazard function on 1000 resamples drawn with replacement from the original cohort. Harrell’s optimism-correcting bootstrap method was used to internally validate the O/E ratio after recalibration.^36^ R software (Version 4.0.2, 2018, R Core Team, Vienna, Austria) was used for statistical analysis. Reporting of this study complied to the Transparent Reporting of a Multivariable Prediction Model for Individual Prognosis or Diagnosis (TRIPOD) statement.^37^

## Results

### Patients

**Figure 1:**
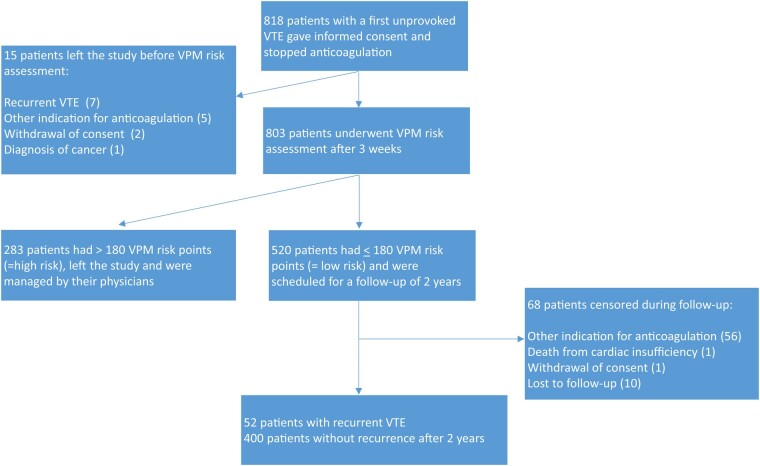
Patient flow

Between January 2013 and May 2019, 818 patients with a first unprovoked DVT of the leg or pulmonary embolism were included (*Figure 1*). Fifteen patients left the study before VPM risk assessment: seven patients had early recurrence, five patients had reasons for indefinite anticoagulation other than VTE, two patients withdrew their consent, and one patient was diagnosed with cancer. The VPM risk assessment was performed in 803 patients 3 weeks after oral anticoagulation had been stopped. A total of 283 patients (35%) with a VPM risk score of more than 180 points (corresponding to a 1-year recurrence risk of more than 5.5%) were classified as high-risk patients and were further managed by their physician. Characteristics of the 520 patients with a low risk of recurrence based on a VPM risk score of 180 points or less are shown in *Table 1*. They were followed for a median of 23.9 months. Their median age was 52 years and 56% were male. A total of 88 patients (17%) had isolated distal DVT, 206 patients (40%) had proximal DVT, and 226 patients (43%) had pulmonary embolism. A total of 441 patients (85%) had been treated with a direct oral anticoagulant for their incident VTE. In 68 patients, follow-up ended prematurely: restart of anticoagulation for other reasons (56 patients), death from cardiac failure (1 patient), withdrawal of consent (1 patient), and lost-to-follow-up (10 patients). These patients were included in the analysis as censored observations.

**Table 1:** Baseline characteristics of 520 patients with a first unprovoked venous thromboembolism and a low recurrence risk as estimated by the Vienna Prediction Model

Characteristic	Patients
Age—years, median (interquartile range)	52 (42, 65)
Sex, number (%)	
Male	289 (56)
Female	231 (44)
D-dimer—ng/mL, median (interquartile range)	280 (190, 450)
Location of incident event, number (%)	
Isolated distal deep-vein thrombosis^a^	88 (17)
Proximal deep-vein thrombosis	206 (40)
Pulmonary embolism	226 (43)
Type of anticoagulant, number (%)	
Vitamin K antagonist	79 (15)
Direct oral anticoagulation	441 (85)
Duration of anticoagulation—months, median (interquartile range)	3.9 (3.3, 5.7)
Follow-up, median (interquartile range)	23.9 (23.8, 23.9)

^a^Defined as a thrombus in the tibial anterior, peroneal, or tibial posterior veins.

### Recurrent venous thromboembolism

**Figure 2:**
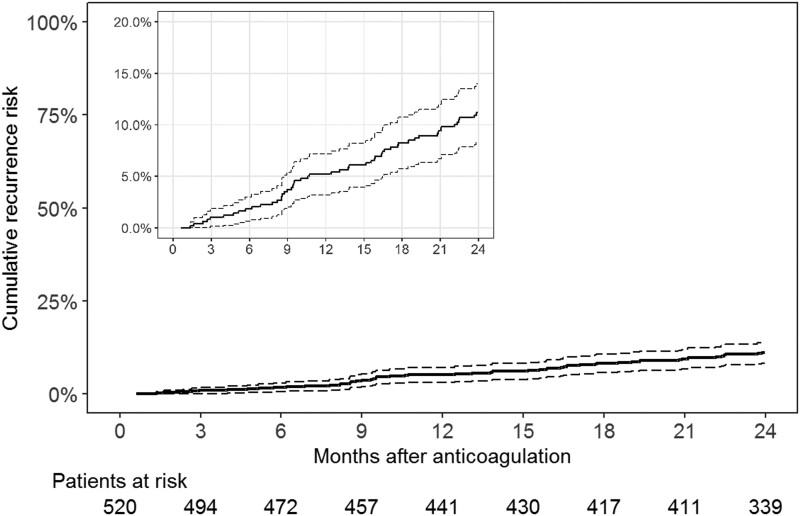
Cumulative probability of recurrent venous thromboembolism. Dashed lines indicate 95% confidence intervals

Fifty-two of the 520 patients had recurrent VTE (*Table 2*). Seven patients (13%) had symptomatic isolated distal DVT, 17 patients (33%) had symptomatic proximal DVT, and 28 patients (54%) had symptomatic pulmonary embolism. Recurrence was unprovoked in 45 patients (87%) and was associated with a transient risk factor in 7 patients (13%; trauma in 3 patients, surgery in 2 patients, and acute medical illness in 2 patients). Of the 20 patients with DVT as index event, 14 had a DVT and 6 had pulmonary embolism as recurrence. Of the 32 patients with a pulmonary embolism as index event, 10 patients had a DVT and 22 patients had pulmonary embolism as recurrence. The cumulative recurrence risk after 12 and 24 months was 5.2% (95% CI 3.2–7.2) and 11.2% (95% CI 8.3–14.0) (*Figure 2*), respectively. Recurrence occurred in the first year with an incidence rate of 5.3 events per 100 patient-years (95% CI 3.4–7.8) and in the second year with an incidence rate of 6.5 events per 100 patient-years (95% CI 4.3–9.4).

**Table 2:** Characteristics of 52 patients with recurrence after a first unprovoked venous thromboembolism

Patient characteristics	Value
Age—years, median (interquartile range)	54 (40–66)
Sex, number (%)	
Male	30 (58)
Female	22 (42)
D-dimer—ng/mL, median (interquartile range)	240 (190–475)
Location of index venous thromboembolism, number (%)	
Proximal deep-vein thrombosis	17 (32)
Isolated distal deep-vein thrombosis	3 (6)
Pulmonary embolism	32 (62)
Location of recurrence, number (%)	
Isolated distal deep-vein thrombosis	7 (13)
Proximal deep-vein thrombosis	17 (33)
Pulmonary embolism	28 (54)
Fatal	0 (0)
Provoking factor, number (%)	
Absent	45 (87)
Present	7 (13)
Type of anticoagulant, number (%)	
Vitamin K antagonist	11 (21)
Direct oral anticoagulant	41 (79)
Duration of anticoagulation—months, median (interquartile range)	3.5 (3.2–5.9)

### Calibration of the Vienna Prediction Model

The 1-year cumulative incidence of recurrence was significantly lower than the threshold value of 8% set in our null hypothesis (*P* = .003). The 2-year cumulative incidence was not significantly lower than the pre-specified threshold value of 13% (*P* = .11). Calibration comparing the risk of recurrent VTE predicted by the VPM with the observed recurrence rate was adequate at 1 year. At 2 years, calibration was adequate for patients with a predicted risk below 5%, while the VPM underestimated the recurrence risk above that value. The calibration slope, ideally at 1.0, was estimated at 1.3 (95% CI 0.3–2.4). The ratio of observed and expected events (O/E ratio), equal to 52/32 = 1.6 (95% CI 1.2–2.0), indicated general underestimation of recurrence probabilities.

### Updating of the Vienna Prediction Model

**Figure 3:**
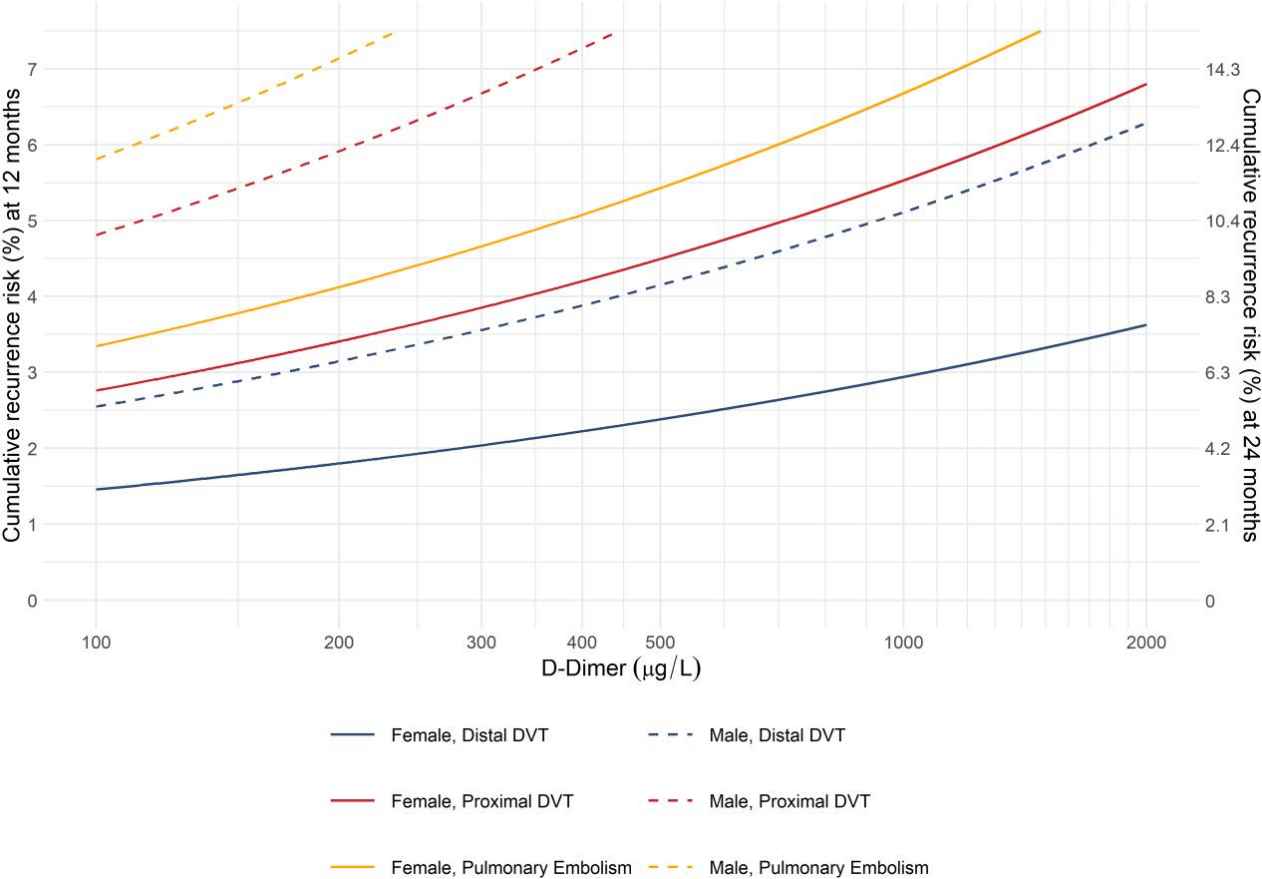
Predicted risk of recurrent venous thromboembolism by the recalibrated Vienna Prediction Model

To address the VPM’s apparent underestimation of the recurrence risk, we first evaluated whether the risk predictors (sex, VTE location, and D-dimer) require adjustment. There was no evidence of lack of fit of the predictor weights (*P* = .32). In a post-hoc analysis, we recalibrated the model by re-estimating the baseline hazard function. Internal bootstrap validation of this recalibration step indicated an O/E ratio of 1.03 (95% CI 0.55–1.74) of the updated VPM. The recurrence risks predicted by the updated VPM for men and women according to the location of the index VTE and D-dimer are depicted in *Figure 3*. It was highest in men with proximal DVT or pulmonary embolism and lowest in women with isolated distal DVT. An intermediate risk was found in women with proximal DVT or pulmonary embolism and in men with isolated distal DVT. In all groups, the recurrence risk increased log-linearly with the D-dimer level. The recurrence risk can be calculated using a web-based calculator (https://clinicalbiometrics.shinyapps.io/VPM_lowrisk/).

## Discussion

Patients with unprovoked VTE have a high risk of recurrence, which is why expert panels suggest extended-phase anticoagulation.^17–19^ However, many patients will never experience a recurrent event but are exposed to a bleeding risk and other burdens of long-term anticoagulation. Hence, we set out to evaluate whether the VPM can identify this subset of low-risk patients in whom limited duration of anticoagulation might be considered. We only followed patients with a predicted 1-year recurrence risk of ≤5.5%. In this set of patients, the cumulative recurrence risk after 1 year was 5.2% and thus low (*Structured Graphical Abstract*). We found that the VPM underestimated the recurrence risk of patients who had a 2-year recurrence rate of more than 5% (*Structured Graphical Abstract*). Poorer model performance in a new set of patients was to be expected and can be explained by a different case mix, change in patient care including novel treatments, as well as by more sensitive diagnostic tools, such as the development of high-resolution imaging techniques for diagnosis of pulmonary embolism.^38^ We did not find strong evidence that the weight of the predictors comprising sex, VTE location, and D-dimer required modification and recalibrated only the baseline hazard. The updated VPM stratifies patients into distinct subgroups according to their recurrence risk (*Figure 3*). Women with a low to moderate D-dimer and men with a low D-dimer are within a lower risk category regardless of thrombosis site, while men with a pulmonary embolism or proximal DVT have a higher recurrence risk. The VPM can be accessed via a web-based risk calculator (https://clinicalbiometrics.shinyapps.io/VPM_lowrisk/).

Our findings complement those of Geersing *et al.*^31^ who studied the performance of the VPM in a randomized controlled trial. A major difference between VISTA and our study is that in VISTA patients with unprovoked VTE were included regardless of their recurrence risk, while we studied only patients with a predicted low recurrence risk. In the subset of VISTA patients with a predicted low 1-year recurrence risk (2%–4%), the observed risk of recurrence was indeed low (2.5%). This finding is in agreement with our observation that the VPM exhibits good discriminative power and good calibration in patients at the lower spectrum of the predicted risk.

Our observation of a low recurrence risk among women is in line with findings of a prospective management study evaluating the HERDOO2 rule.^39^ According to that rule, women with a first unprovoked VTE event and none or only one of the HERDOO2 criteria (signs of post-thrombotic syndrome, of a high D-dimer, and of a high body mass index or at younger age) have a low risk of recurrent VTE, while no subgroup of men could be identified at low recurrence risk.

To establish the optimal duration of anticoagulation, the individual risk of recurrent VTE must be weighed against the individual bleeding risk also considering the respective case fatality rates. A meta-analysis showed an incidence of major bleeding during extended-phase anticoagulation of 1.68 per 100 patient-years with incidence rates per 100 person-years for vitamin K antagonists of 2.00 (95% CI 1.56–2.50) and for direct oral anticoagulants of 1.20 (95% CI 0.74–1.77).^20^ The case fatality rate of major bleeding was 8.4% and thus twice that of recurrent VTE.^16^ In a prospective cohort study of 839 patients, we even recorded a lower incidence of fatal pulmonary embolism with 4 out of 263 patients with recurrence.^15^ Besides bleeding, extended-phase anticoagulation can be associated with inconveniences in lifestyle, work conditions, and leisure activities causing a limitation of patient satisfaction.

Thus, patients and their physicians may well have their own, individual assessment on the balance of risks of recurrence and bleeding which could deviate from that suggested by guidelines. For instance, patients with a higher bleeding risk may accept a somewhat higher risk of recurrent VTE. Estimation of the recurrence risk by the VPM could also support informed decision-making when the balance between risks and benefits is uncertain or in patients who are undecided or even reluctant towards indefinite anticoagulation.

When applying the VPM, anticoagulation needs to be stopped shortly before D-dimer measurement which carries a risk of early recurrent VTE. Indeed, in our study, a very small proportion of patients had recurrent VTE within 3 weeks after stopping anticoagulation. For these patients, an obvious and clinically meaningful pattern of patient characteristics and risk factors was not identifiable. This small risk of early recurrence must be balanced against the benefit of identifying patients at low recurrence risk in whom limited duration of anticoagulation might be justified. Clearly, this feature of the VPM must be discussed with the patients. Bridging with a platelet function inhibitor might reduce the recurrence risk during this period, but its effect on the performance of the VPM is unclear. Notably, except for the HERDOO2 rule, D-dimer is measured after anticoagulation in all other prediction models.

The recurrence risk of patients with unprovoked distal DVT is less well studied, as often also patients with provoked DVT or muscle vein thrombosis were included.^40,41^ We included only patients who had unprovoked symptomatic thrombosis in the tibial anterior, peroneal, and/or tibial posterior veins. In this subset of patients, we and others recorded a 10-year recurrence risk of about 20% with a considerable proportion of patients with pulmonary embolism at recurrence.^15,42^ Regarding the duration of anticoagulation, guidelines do not differentiate between patients with isolated distal or proximal DVT.^17,18^ Whereas women with an isolated distal DVT in our study had a very low recurrence risk, we recorded a higher risk in men which was in the range of that of women with proximal DVT. We believe that assessment of the risk of recurrence in patients with isolated distal DVT could be clinically relevant and is a valuable feature of our model.

Our study has several strengths. We studied a homogenous cohort of patients by excluding patients with a history of VTE before the index event and patients with VTE provoked by a transient risk factor including oestrogen use.^7^ The proportion of patients within the low-risk category as identified by the VPM was as high as 65% of the total population with unprovoked VTE. We performed a baseline compression ultrasound to facilitate the diagnosis of recurrence according to an algorithm.^32^ Patients were seen in person, and few were lost to follow-up. Most patients were treated with a direct oral anticoagulant for their first VTE rather than with a vitamin K antagonist.

Our study has limitations. We did not screen for laboratory thrombophilia but excluded patients with already documented major thrombophilia. We included few patients with another ethnicity than Caucasian. We did not evaluate the recurrence risk in patients with a VPM risk score of more than 180 risk points, which corresponds to a 1-year recurrence risk of more than 5.5%. From our view, withdrawal of anticoagulation is not justified in these high-risk patients. The recalibrated VPM should preferably be externally validated.

In this prospective evaluation of the performance of the VPM, the 1-year rate of recurrent VTE was 5.2%, which may be regarded by some as too high as to withhold anticoagulation. Recalibration improved the performance, and the VPM identifies patients with a first unprovoked VTE and a lower risk of recurrence and further stratifies them into different low-risk subcategories. Such a personalized risk assessment could facilitate informed decision-making on the duration of secondary thromboprophylaxis when the optimal balance between risks and benefits is uncertain.

## Supplementary data

Supplementary data are not available at *European Heart Journal* online.

## Data Availability

The data underlying this article are available from the corresponding author upon reasonable request.

## Pre-registered Clinical Trial Number

The pre-registered clinical trial number is NCT01972243.
